# Analysis of synonymous codon usage in Hepatitis A virus

**DOI:** 10.1186/1743-422X-8-174

**Published:** 2011-04-16

**Authors:** Yiqiang Zhang, Yongsheng Liu, Wenqian Liu, Jianhua Zhou, Haotai Chen, Yin Wang, Lina Ma, Yaozhong Ding, Jie Zhang

**Affiliations:** 1 State Key Laboratory of Veterinary Etiological Biology, Lanzhou Veterinary Research Institute, Chinese Academy of Agricultural Sciences, Lanzhou 730046, Gansu, China; 2 Laboratory of Animal Quarantine, College of Animal Medical, Sichuan Agricultural University, Ya'an 625014, Sichuan, China

## Abstract

**Background:**

Hepatitis A virus is the causative agent of type A viral hepatitis, which causes occasional acute hepatitis. Nevertheless, little information about synonymous codon usage pattern of HAV genome in the process of its evolution is available. In this study, the key genetic determinants of codon usage in HAV were examined.

**Results:**

The overall extent of codon usage bias in HAV is high in *Picornaviridae*. And the patterns of synonymous codon usage are quite different in HAV genomes from different location. The base composition is closely correlated with codon usage bias. Furthermore, the most important determinant that results in such a high codon bias in HAV is mutation pressure rather than natural selection.

**Conclusions:**

HAV presents a higher codon usage bias than other members of *Picornaviridae*. Compositional constraint is a significant element that influences the variation of synonymous codon usage in HAV genome. Besides, mutation pressure is supposed to be the major factor shaping the hyperendemic codon usage pattern of HAV.

## Background

Hepatitis A virus (HAV), the causative agent of type A viral hepatitis, is an ancient human virus that was first identified in the stools of infected people in 1973 [1]. HAV is a non-enveloped, single-stranded positive-sence RNA virus which belongs to order *Picornavirales*, family *Picornaviridae*, the genus *Hepatovirus *in virus taxonomy [2-4]. The genome of HAV is approximately 7500 nucleotide in length and contains a large open-reading frame (ORF) encoding a polyprotein in which the major capsid proteins represent the amino-terminal third, with the remainder of the polyprotein comprising a series of nonstructural proteins required for HAV RNA replication: 2B, 2C, 3A, 3B, 3C^pro ^and 3D^pol^. Based on the studies of genetics, HAV was proposed to divide into six different genotypes [5]. However, there is only one known serological group of human HAV [6,7]. Although HAV causes occasional, dramatic disease outbreaks of acute hepatitis with fatal outcomes in otherwise healthy adults as well as isolated severe cases of hepatitis, it has never been associated with chronic liver disease [8].

As we all know, the genetic code chooses 64 codons to represent 20 standard amino acids and stop signals. These alternative codons for the same amino acid are termed as synonymous codons. Synonymous mutations tend to occur in the third base position, but the cases can be interchanged without altering the primary sequence of the polypeptide product. Some reports indicate that synonymous codons are not chosen equally both within and between genomes [9-13]. In general, codon usage variation may be the product of natural selection and/or mutation pressure for accurate and efficient translation in various organisms [14-21]. It is well known that codon usage variation is considered as an indicator of the forces shaping genome evolution. In addition, compared with natural selection, mutation pressure plays an important role in synonymous codon usage pattern in some RNA viruses [18,22,23].

Nevertheless, little information about codon usage pattern of HAV genome including the relative synonymous codon usage (RSCU) and codon usage bias (CUB) in the process of its evolution is available. In this study, the key genetic determinants of codon usage index in HAV were examined.

## Results

### Synonymous codon usage in HAV

The values of nucleotide contents in complete coding region of all 21 HAV genomes were analyzed (Table 1). Evidently, (C+G)% content fluctuated from 36.9 to 37.9, with a mean value of 37.15 and S.D of 0.28, indicating that nucleotides A and U were the major elements of HAV genome. Comparing the values of A_3_%, U_3_%, C_3_% and G_3_%, it is clear that U_3_% was distinctly high, and C_3_% was the lowest of all. The (C_3_+G_3_)% in complete coding region of each HAV genome fluctuated from 28.8 to 31.5, with a mean value of 29.92 and S.D of 0.62. And the effective number of codons (ENC) values of these HAV genomes fluctuated from 38.8 to 40.7, with a mean value of 39.34 and S.D. of 0.58. The ENC values for these HAV genomes were a little low indicating that the there is a particular extent of codon preference in HAV genome. The details of the overall relative synonymous codon usage (RSCU) values of 59 codons in 21 HAV genomes were analyzed (Table 2). Most preferentially used codons in HAV are A-ended or U-ended codons except the Gln and Leu whose optimized codons are CAG and UUG ending by G, respectively. Interestingly, HAV prefers U-ended optimized codons to A-ended codons.

**Table 1:** Identified nucleotide contents in complete coding region (length >250 bps) in hepatitis A virus (21 isolates) genome

SN	A%	**A**_**3**_**%**	U%	**U**_**3**_**%**	C%	**C**_**3**_**%**	G%	**G**_**3**_**%**	(C+G)%	**(C**_**3**_**+G**_**3**_**)%**	^**a**^**ENC**
1	29.8	26.9	32.6	41.9	15.3	9.5	22.3	21.7	37.6	31.2	39.6
2	29.9	27.4	33.0	43.2	15.2	9.0	21.9	20.4	37.1	29.4	39.2
3	30.2	27.7	32.9	43.4	15.3	9.2	21.6	19.6	36.9	28.8	39.2
4	30.0	27.2	32.9	43.1	15.3	9.3	21.8	20.4	37.1	29.7	39.0
5	30.2	27.9	32.7	42.1	15.5	10.0	21.6	20.0	37.1	30.0	38.9
6	30.1	27.7	32.9	42.9	15.2	9.3	21.8	20.2	36.9	29.5	38.8
7	30.2	28.0	32.7	42.1	15.4	10.0	21.6	19.9	37.0	29.9	38.9
8	30.3	28.1	32.7	42.1	15.5	10.0	21.5	19.8	36.9	29.8	39.0
9	30.2	28.0	32.7	42.1	15.4	10.0	21.6	19.9	37.0	29.9	38.9
10	30.1	27.4	32.9	42.8	15.2	9.2	21.8	20.6	37.0	29.8	38.9
11	29.8	27.0	32.4	41.9	15.8	10.3	22.0	20.7	37.7	31.0	40.7
12	30.3	27.9	32.8	42.6	15.3	9.4	21.6	20.1	36.9	29.5	38.8
13	29.6	25.8	32.5	42.7	16.1	11.0	21.8	20.5	37.9	31.5	40.7
14	29.8	26.7	32.7	43.3	15.9	10.3	21.5	19.7	37.4	30.0	40.0
15	30.1	27.5	32.9	43.3	15.3	9.1	21.7	20.2	37.0	29.3	39.2
16	30.0	27.4	32.7	42.5	15.6	10.1	21.7	20.1	37.3	30.2	40.0
17	30.0	27.3	32.6	42.6	15.5	9.6	21.8	20.4	37.3	30.0	39.6
18	30.1	27.5	32.7	42.7	15.4	9.7	21.7	20.1	37.1	29.8	39.5
19	30.3	28.0	32.6	42.0	15.5	10.0	21.6	19.9	37.0	29.9	38.9
20	30.1	27.5	32.9	42.9	15.2	9.1	21.8	20.6	37.0	29.7	39.1
21	30.0	27.4	32.9	42.8	15.3	9.2	21.8	20.6	37.0	29.8	39.2

^a^ENC is effective number of codons

**Table 2:** Synonymous codon usage of the whole coding sequence in hepatitis A virus

^**a**^**AA**	Codon	^**b**^**RSCU**	AA	Codon	RSCU
Ala	GCA	1.25	Leu	CUA	0.22
	GCC	0.59		CUC	0.20
	GCG	0.02		CUG	0.64
	**GCU**	**2.12**		CUU	1.12
Arg	**AGA**	**4.31**		UUA	1.29
	AGG	1.24		**UUG**	**2.49**
	CGA	0.13	Lys	**AAA**	**1.26**
	CGC	0.11		AAG	0.73
	CGG	0.02	Phe	UUC	0.43
	CGU	0.15		**UUU**	**1.56**
Asn	AAC	0.37	Pro	CCA	1.61
	**AAU**	**1.62**		CCC	0.41
Asp	GAC	0.32		CCG	0.05
	**GAU**	**1.67**		**CCU**	**1.91**
Cys	UGC	0.36	Ser	AGC	0.11
	**UGU**	**1.63**		AGU	0.74
Gln	CAA	0.94		UCA	2.03
	**CAG**	**1.05**		UCC	0.66
Glu	**GAA**	**1.12**		UCG	0.11
	GAG	0.87		**UCU**	**2.33**
Gly	**GGA**	**1.78**	Thr	ACA	1.71
	GGC	0.49		ACC	0.36
	GGG	0.62		ACG	0.10
	GGU	1.10		**ACU**	**1.81**
His	CAC	0.33	Tyr	UAC	0.42
	**CAU**	**1.66**		**UAU**	**1.57**
Ile	AUA	0.64	Val	GUA	0.39
	AUC	0.32		GUC	0.31
	**AUU**	**2.03**		GUG	1.04
				**GUU**	**2.24**

^a^AA is the abbreviation of amino acid.

^b^RSCU values are mean values

^c^The preferentially used codons for each amino acid are described in bold.

### Correspondence analysis (COA)

**Figure 1:**
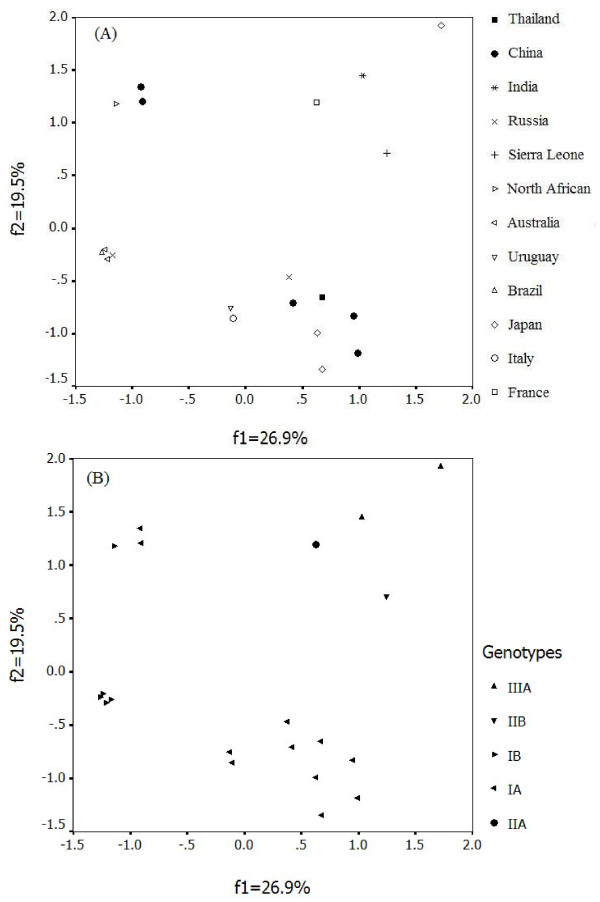
**A plot of value of the first and second axis of each complete coding region in COA**. The first axis (ƒ'_1_) accounts for 26.98% of the total variation, and the second axis (ƒ'_2_) accounts for 19.50% of the total variation. A): Each HAV complete coding region was divided by geographical area. B): Each HAV complete coding region was divided by genotype.

To investigate the major trend in codon usage variation among HAV, COA was used for all 21 HAV complete coding regions selected for this study. COA detected one major trend in the first axis (ƒ'_1_) which accounted for 26.98% of the total variation, and another major trend in the second axis (ƒ'_2_) which accounted for 19.50% of the total variation. A plot of the first and second principal axes of the complete coding region of each gene was shown in Figure 1. It is clear that coordinate of each gene is relatively isolate except the Australia isolates, Brazil isolate and one Russia isolate. Nevertheless, these relatively isolated spots tend to cluster into several groups according to the same genotype. But MBB which isolated from North Africa had a special codon usage pattern contrasting with the other IB strains. All above imply that these strains of HAV isolated from different places, even the same genotype, have different trend in codon usage variation. Interestingly, the pattern of codon usage in vaccine strain H2 change to MBB-like pattern after continuous culturing in a human diploid cell line (KMB17), *i.e. *H2K5 and H2K20, suggesting that host was an element that could dramatically influence the codon usage pattern.

### Compositional properties of HAV genomes

In order to analyze whether the codon usage variation of HAV genome was regulated by natural selection or mutation pressure, the A%, U%, C%, G% and (C+G)% were respectively compared with A_3_%, U_3_%, C_3_%, G_3_% and (C_3_+G_3_)% (Table 3). There was a complex correlation existing in nucleotide compositions. In detail, A_3_%, C_3_% and G_3_% have a significant negative correlation with C%, U% and A%, respectively. These data suggest that the nucleotide constraint may influence synonymous codon usage. However, A_3_% has non-correlation with U%, and U_3_% has non-correlation with A%, C%, G% and (C+G)%, respectively, which haven't indicated any peculiarity about synonymous codon usage. Furthermore, C_3_% and G_3_% have non-correlation with G% and C% respectively, indicating these data probably don't reflect the true feature of synonymous codon usage as well. Therefore, linear regression analysis was implemented to analyze the correlation between synonymous codon usage bias and nucleotide compositions. Details of correlation analysis between the first two principle axes (ƒ'_1 _and ƒ'_2_) of each HAV genome in COA and nucleotide contents were analyzed (Table 4). In surprise, only A_3_% has a significant correlation with both principle axes which represent the major trend in codon usage variation, suggesting that nucleotide A is the major factor influencing the synonymous codon usage pattern of HAV genome. However, interestingly, although the (ƒ'_2_) value has non-correlation with base nucleotide C and G contents on the third codon position respectively, it is observably related to (C_3_+G_3_)%, suggesting that codon usage patterns in HAV probably be correlated with (C_3_+G_3_)% to a specific extent. Overall, compositional constraint is a factor shaping the pattern of synonymous codon usage in HAV genome.

**Table 3:** Summary of correlation analysis between the A, U, C, G contents and A_3_, U_3_, C_3_, G_3 _contents in all selected samples.

	**A**_**3**_**%**	**U**_**3**_**%**	**C**_**3**_**%**	**G**_**3**_**%**	**(C**_**3**_**+G**_**3**_**)%**
A%	r = 0.965**	r = -0.160^NS^	r = -0.328^NS^	r = -0.555**	r = -0.679**
U%	r = 0.357^NS^	r = 0.691**	r = -0.853**	r = -0.164^NS^	r = -0.825**
C%	r = -0.622**	r = -0.191^NS^	r = 0.926**	r = -0.140^NS^	r = 0.662**
G%	r = -0.532*	r = -0.181^NS^	r = -0.139^NS^	r = 0.945**	r = 0.580**
(C+G)%	r = -0.844**	r = -0.270^NS^	r = 0.687**	r = 0.462*	r = 0.907**

^a^r value in this table is calculated in each correlation analysis.

NS means non-significant (p > 0.05).

* means 0.01 < p < 0.05

**means p < 0.01

**Table 4:** Analysis of correlation between the first two principle axes and nucleotide contents in samples.

Base compositions	***f***_***1***_***' *(26.98%)**	***f***_***2***_***' *(19.50%)**
A_3_%	r = -0.714**	r = -0.573**
U_3_%	r = 0.302^NS^	r = 0.151^NS^
C_3_%	r = 0.274^NS^	r = 0.332^NS^
G_3_%	r = 0.178^NS^	r = 0.433*
(C_3_+G_3_)%	r = 0.361^NS^	r = 0.589**

^a^r value in this table is calculated in each correlation analysis.

NS means non-significant.

* means 0.01 < p < 0.05

**means p < 0.01

### Mutational bias is another main factor leading to codon usage variation

**Figure 2:**
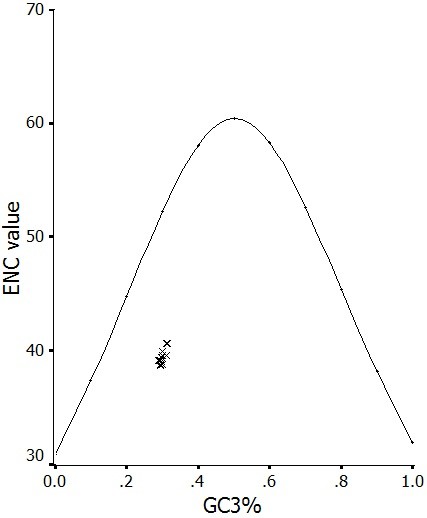
**Effective number of codons used in each ORF plotted against the GC3s**. The continuous curve plots the relationship between GC3s and ENC in the absence of selection. All of spots lie below the expected curve.

ENC-plot was considered as a part of the general strategy to investigate patterns of synonymous codon usage. The ENC-plots of the genes, whose codon choice is constrained only by a C_3_+G_3 _composition, will lie on or just below the curve of the predicted values (Wright, 1990). ENC values of each HAV genome were plotted against its corresponding (C_3_+G_3_)%. All of the spots lie below the curve of the predicted values, as shown in Figure 2, suggesting that the codon usage bias in all these 21 HAV genomes is principally influenced by the mutational bias.

## Discussion

Overtime, there have been more and more features that are unique to HAV within the family *Picornaviridae*, including its tissue tropism, its virion morphogenesis, its genetic distance from other members of this family, the important details of the processing of the viral polyprotein and the interactions of the virus with host cells [24]. After we analyzed synonymous codon usage in HAV (Table 2), we found that comparing with other viruses of *Picornaviridae*, such as Coxsackievirus A9 (ENC = 55.6), Enterovirus 71 (ENC = 56.6), Poliovirus type 3 (ENC = 54.2), Rhinovirus type 89 (ENC = 45.9) [23] and Food-and-Mouth Disease virus (mean ENC = 51.53) [21], the ENC values for HAV are a little low (mean ENC = 39.34). Although the ENC values for Coxsackievirus, Enterovirus, Poliovirus and Rhinovirus are not the mean value, it is also suggesting that the overall extent of codon usage bias in HAV genomes is rather high in *Picornaviridae*. In fact, Sánchez et al. have previously reported that HAV presents a higher codon usage bias than other members of the family, which conveys in the adaptation to use abundant and rare codons [25]. As a result, HAV codon usage has evolved to be complementary to that of human cells, never adopting codons those abundant for the host cell, even in some instances using these abundant codons as rare codons [26].

Since the variation and evolution of virus generally appear in the changes of virus genome composition, compositional constraint was assumed to be closely correlated with the synonymous codon usage pattern [18,19,27-30]. Nucleotide U content was the highest, and the ratio of U_3_% was much higher than the other base composition on the third codon position (Table 3), which interpreted why most of the preferentially used codons are U-ended codons (Table 2). Despite the ratio of U_3_% was the highest, the major compositional constraint, which shaping the synonymous codon usage pattern of HAV genome, was from the percent of nucleotide A on the third codon position (Table 4). Moreover, two principle axes (ƒ'_1 _and ƒ'_2_) are not correlated with the other base compositions except nucleotide A (Table 4). This discovery was different from many reports which suggest that C+G compositional constraints were the major factor influencing codon usage bias in virus genome [18,29,30]. Therefore, we supposed that the compositional constraint was from not only C+G contents but also A and/or U contents. In addition, we found that A_3_% has a remarkable correlation with (C+G)% (Table 3). Hence, we could infer that A_3_% could influence the synonymous codon usage pattern through coordinating the contents of (C+G)%. Moreover, each composition was closely correlated with one of the other compositions, and each composition has a striking negative correlation with the other compositions. The (C_3_+G_3_)% was correlated with all the base compositions especially U and C contents. All these data suggest that there were kinds of complex and fantastic interrelations existing among these base compositions to regulate the codon usage bias. In brief, compositional constraint can indeed determine the variation of synonymous codon usage in virus genome.

Mutational pressure and natural selection are generally thought to be the main factors that account for codon usage variation between genes in different organisms [14-21]. We wished to determine which should be responsible for the extreme codon usage bias in HAV. In the present study, the mutational pressure was determined to be the more important factor for the codon usage bias in HAV, which is shown in Figure 2, indicating that the codon usage in HAV genome is influenced by the C+G content which is usually assumed to be the result of mutational pressure. Actually, it is previously reported that mutation pressure rather than natural selection is the most important determinant of the codon bias in human RNA viruses [23]. Since mutation rates in RNA viruses are much higher than those in DNA viruses [31], it is understandable that mutational pressure is the major factor of shaping codon usage pattern in the 21 HAV strains included in our study. Despite this, HAV does not appear to undergo the rapid accumulation of genetic changes seen in many RNA viruses. Because HAV exploits a very low translation rate and a very low replication rate to promote and ensure its survival [26,32], it shows a quite low mutation rate than other members of the family *Picornaviridae *[24,33].

Since HAV mutation rate is much lower than other members of the family *Picornaviridae*, how does it form such a higher codon usage bias than other members of the family? Furthermore, how does it form kinds of trends in codon usage variation among different stains (Shown in Figure 1) in the condition of the similar nucleotide contents (Table 2)? This could be ascribed to the distinct endemicity of HAV, which is speculated from the result of COA. Early comparative studies of the nucleotide sequences of different human HAV strain suggested that sequence correlation could be correlated with the geographical origin of viruses [34,35]. It is well known that quasispecies dynamics is characterized by continuous generation of variant viral genomes, competition among them, and selection of the fittest mutant distributions in any given environment. As other RNA viruses, HAV exists in vivo as distributions of closely related variant referred to as quasispecies [25,32]. HAV strains maintained their low rate of accumulating mutations over a long period of time so that it developed specific ecological niches [33]. Because of surviving in different geographical area, different human race and different rounds of replication, the extreme codon usage bias of HAV was established over a long time. Moreover, in the context of a very low mutation rate, the extreme codon usage bias of HAV was conserved so that a distinct endemicity was generated.

## Conclusions

HAV presents a higher codon usage bias than other members of *Picornaviridae*. The most important determinant of the high codon bias in HAV is mutation pressure which is also the main element shaping the hyperendemic codon usage pattern of HAV despite the mutation rate of HAV is quite low. Besides, compositional constraint is another factor influencing the synonymous codon usage in HAV. Although basic knowledge of codon usage patterns of HAV and the factors regulating the synonymous codon usage are demonstrated in our present study, more comprehensive analysis is necessary for revealing the deeper characteristic of synonymous codon usage in HAV genome.

## Materials and methods

### Sequences

The 21 available complete RNA sequences of HAV were obtained from GenBank randomly in October 2010. The serial number (SN), GenBank number, genotype and other detail information are listed in Table 5.

**Table 5:** Information of hepatitis A virus genomes used in this study

SN	Strain	Genotype	Location	Accession No.
1	CF53/Berne	IIA	France	AY644676.1
2	F.G.	IA	Italy	X83302.1
3	FH2	IA	Japan	AB020568.1
4	FH3	IA	Japan	AB020569.1
5	HAF-203	IB	Brazil	AF268396.1
6	HAV5	IA	Uruguay	EU131373.1
7	HAVgs1	IB	Derived from HM-175	NC_001489.1
8	HM-175	IB	Australia	M16632.1
9	HM-175wp	IB	Australia	M14707.1
10	MBB	IB	North Africa	M20273.1
11	SLF88	IIB	Sierra Leone	AY644670.1
12	VBA-07	IA	Russia	EU251188.1
13	HA-JNG04-90	IIIA	Japan	AB279732.1
14	PN-IND	IIIA	India	EU011791.1
15	H2	Vaccine strain IA	China	EF406357.1
16	Lu38/WT	IA	China	AF357222.1
17	LY6	IA	China	AF485328.1
18	LP014	IA	Thailand	EF207320.1
19	IVA	IB	Russia	DQ646426.1
20	H2K20	Derived from H2	China	EF406361.1
21	H2K5	Derived from H2	China	AY644676.1

### Measures of relative synonymous codon usage

Relative synonymous codon usage values of each codon in a gene were calculated to investigate the characteristics of synonymous codon usage without the confounding influence of amino acid composition of different gene sample [14]. The RSCU value of the *i*th codon for the *j*th amino acid was calculated as:

Where g_ij _is the observed number of the *i*th codon for *j*th amino acid which has n_i _type of synonymous codons. When the codon with RSCU values close to 1.0, it means that this codon is chosen equally and randomly.

The ENC was calculated to quantify the codon usage bias of an ORF [36], which is the best estimator of absolute synonymous codon usage bias [37]. The larger extent of codon preference in a gene, the smaller the ENC value is. And the index GC3s was used to calculate the fraction of the nucleotides G+C at the synonymous third codon position (excluding Met, Trp, and the termination codons).

### Correspondence analysis

Multivariate statistical analysis can be used to explore the relationships between variables and samples. In this study, correspondence analysis was used to investigate the major trend in codon usage variation among genes. In this study, the complete coding region of each gene was represented as a 59 dimensional vector, and each dimension corresponds to the RSCU value of one sense codon (excluding Met, Trp, and the termination codons) [38].

### Correlation analysis

Correlation analysis was used to identify the relationship between nucleotide composition and synonymous codon usage pattern [39]. This analysis was implemented based on the Spearman's rank correlation analysis way.

All statistical processes were carried out by with statistical software SPSS 11.5 for windows.

## Competing interests

The authors declare that they have no competing interests.

## Authors' contributions

YQZ conceived of the study, downloaded these sequences, calculated the data, analyzed the results and drafted the manuscript; YSL conceived of the study, supervised the research, analyzed the results and helped draft the manuscript; JHZ calculated and visualized the data; WQL, HTC, YW, LNM and YZD assisted with data analysis; JZ supervised the research and helped draft the manuscript. All authors read and approved the final manuscript.
